# Urinary Extracellular Vesicles as a Source of NGAL for Diabetic Kidney Disease Evaluation in Children and Adolescents With Type 1 Diabetes Mellitus

**DOI:** 10.3389/fendo.2021.654269

**Published:** 2022-01-03

**Authors:** Francisca Ugarte, Daniela Santapau, Vivian Gallardo, Carolina Garfias, Anahí Yizmeyián, Soledad Villanueva, Carolina Sepúlveda, Jocelyn Rocco, Consuelo Pasten, Cinthya Urquidi, Gabriel Cavada, Pamela San Martin, Francisco Cano, Carlos E. Irarrázabal

**Affiliations:** ^1^ Pediatric Endocrinology Unit, Pediatric Service, Clinica Universidad de los Andes, Santiago, Chile; ^2^ Departament of Pediatrics, Facultad de Medicina, Universidad de los Andes, Santiago, Chile; ^3^ Pediatric Endocrinology and Diabetes Unit, Hospital Dr. Exequiel González Cortés, Santiago, Chile; ^4^ Centro de Medicina Regenerativa, Facultad de Medicina, Clinica Alemana-Universidad del Desarrollo, Santiago, Chile; ^5^ Programa de Fisiología, Laboratorio de Fisiología Integrativa y Molecular, Centro de Investigación e Innovación Biomédica (CIIB), Universidad de los Andes, Santiago, Chile; ^6^ School of Medicine, Facultad de Medicina, Universidad de los Andes, Santiago, Chile; ^7^ Department of Epidemiology and Health Studies, Facultad de Medicina, Universidad de los Andes, Santiago, Chile; ^8^ Department of Public Health, School of Public Health, Faculty of Medicine, Universidad de Chile, Santiago, Chile; ^9^ Pediatric Nephrology Unit, Pediatric Service, Hospital Luis Calvo Mackennna, Santiago, Chile

**Keywords:** diabetic kidney disease, type 1 diabetes mellitus, children, NGAL, urinary extracellular vesicles

## Abstract

**Background:**

Tubular damage has a role in Diabetic Kidney Disease (DKD). We evaluated the early tubulointerstitial damage biomarkers in type-1 Diabetes Mellitus (T1DM) pediatric participants and studied the correlation with classical DKD parameters.

**Methods:**

Thirty-four T1DM and fifteen healthy participants were enrolled. Clinical and biochemical parameters [Glomerular filtration Rate (GFR), microalbuminuria (MAU), albumin/creatinine ratio (ACR), and glycated hemoglobin A1c (HbA1c)] were evaluated. Neutrophil gelatinase-associated lipocalin (NGAL), Hypoxia-inducible Factor-1α (HIF-1α), and Nuclear Factor of Activated T-cells-5 (NFAT5) levels were studied in the supernatant (S) and the exosome-like extracellular vesicles (E) fraction from urine samples.

**Results:**

In the T1DM, 12% had MAU >20 mg/L, 6% ACR >30 mg/g, and 88% had eGFR >140 ml/min/1.72 m^2^. NGAL in the S (NGAL-S) or E (NGAL-E) fraction was not detectable in the control. The NGAL-E was more frequent (p = 0.040) and higher (p = 0.002) than NGAL-S in T1DM. The T1DM participants with positive NGAL had higher age (p = 0.03), T1DM evolution (p = 0.03), and serum creatinine (p = 0.003) than negative NGAL. The NGAL-E correlated positively with tanner stage (p = 0.0036), the median levels of HbA1c before enrollment (p = 0.045) and was independent of ACR, MAU, and HbA1c at the enrollment. NFAT5 and HIF-1α levels were not detectable in T1DM or control.

**Conclusion:**

Urinary exosome-like extracellular vesicles could be a new source of early detection of tubular injury biomarkers of DKD in T1DM patients.

## Introduction

Diabetes Mellitus (DM) is one of the most prevalent non-transmissible chronic diseases (1). According to a 2019 International Diabetes Federation, the global prevalence of Diabetes is 463 million people and 578 million people will be affected by 2030 (1). Furthermore, Type 1 DM (T1DM) incidence has increased worldwide over the last decades (2–4).

Despite the advances in diabetes treatment, many patients develop diabetic kidney disease (DKD) (5, 6). DKD is a chronic microvascular complication affecting 30% of patients with type 1 diabetes mellitus (T1DM), 20% of patients with type 2 diabetes mellitus (T2DM), and is the most frequent cause of end-stage renal disease (ESRD), morbidity, and mortality (7, 8). DM is the most frequent cause of ESRD accounting for 50% of cases in the developed world and is significantly associated with increased mortality risk (9). The pathogenesis of DKD is multifactorial (10) and has been classically considered as a glomerular disease. DKD is classified according to the levels of proteinuria and glomerular filtration rate (GFR). Microalbuminuria is currently considered the best predictor of the early stages of DKD (11–14). However, recently the literature has described that tubulointerstitial damage appears in the early stages of DKD, contributing to the progression of renal disease (15–17).

Several biomarkers of tubular damage have been proposed for early diagnosis of acute kidney injury (AKI). Neutrophil gelatinase-associated lipocalin (NGAL*)* is secreted in high amounts into the urine and blood from tubular cells during AKI before serum creatinine rises (18). In T1DM, NGAL concentration is increased in blood and urine samples before the microalbuminuria condition (19). A positive correlation of NGAL with albumin excretion rate (AER) and hemoglobin A1c (HbA1c) has been described (20, 21). In T2DM patients, a high expression of NGAL is a strong predictor of glomerular filtration rate (GFR) impairment (22, 23).

On the other hand, Nuclear Factor of Activated T-cells 5 (NFAT5), a transcriptional factor, is involved in the hypertonicity cellular response (24, 25), renal ischemia, and reperfusion (26, 27), hypoxia (28), and diabetic nephropathy (29). Moreover, the alpha subunit of Hypoxia-inducible Factor 1 (HIF-1*α*), involved in various mechanisms of adaptation and survival of hypoxia, is stimulated under hyperglycemia in diabetic rat models (30, 31), and the diabetic patient has shown a loss of cellular adaptation to hypoxia (32).

Exosomes are small extracellular vesicles (30–200 nm) generated from intracellular endosomes, and they act as mediators in cell-to-cell communication, transferring proteins, lipids, DNA, and RNA species (miRNA, mRNA, and tRNAs). The exosomes contain molecules that belong to different cell types (called cargo) and are a new source of biomarkers of different diseases (33–35).

The study aimed to evaluate the correlation of the tubular kidney damage (NGAL) and cell stress (NFAT5 and HIF-1α) biomarkers present in the urinary extracellular vesicles with classical clinical parameters DKD used in T1DM.

## Material and Methods

### Subjects

The present study is a cross-sectional study. The study group inclusion criteria were children and adolescents with T1DM attended at the Endocrinology and Diabetes Unit at the Dr. Exequiel González Cortés (HEGC) Children’s Hospital in Santiago, Chile, aged 2 to 18 years old, more than 12 months with diabetes, without other chronic systemic or kidney disease, or glucocorticoid medication. 57/154 T1DM were not eligible (7 were >18 years old; 34 had less than 12 months of DM, and 12 did not have regular medical evaluation). There were 101/154 participants who met the inclusion criteria. Thus, 40 were randomly selected using the simple randomized method (STATA). Finally, 34/40 T1DM participants completed the study. The Control group included 15 healthy children and adolescents matched by age and sex attending for minor dermatological surgeries at our hospital or healthy volunteers. The present study had the approbation of the Scientific Ethics Committee from the South Metropolitan Public Health Service (Santiago, Chile). The legal guardian signed the Informed Consent for each participant.

### Anthropometric Assessment

Weight (SECA^®^ scale, 0.1 kg precision) and height (Genentech^®^ stadiometer; 1 mm precision) were measured. The height-for-age Z-score according to the World Health Organization (WHO) parameters in children under six years (36) and the National Center for Health Statistics (NCHS) in patients older than six years (37) were used as criteria. Following the Center for Disease Control (CDC) growth charts, the BMI Z-score was calculated following Center for Disease Control (CDC) growth charts (38). The pubertal stage was examined by a board-certified Pediatric Endocrinologist, according to Tanner (39). Blood pressure measurements (automatic DINAMAP^®^ device) and hypertension (defined as systolic or diastolic blood pressure >95th percentile) for age, sex, and height were taken according to the Fourth Report of High Blood Pressure in Children and adolescents (40).

### Diabetes Assessment

The age at diagnosis and time of evolution with T1DM was recorded. Kidney function was established by serum creatinine measurement (COBAS C-501 (Hitachi), microalbuminuria (MAU), and urinary creatinine (COBAS C-501). Estimated Glomerular filtration rate (eGFR) was calculated by Schwartz-modified equation (0.413 ∗ height/sCr) (41). The eGFR >140 ml/min/1.72 m^2^ was considered hyperfiltration. The MAU >20 mg/L (42) and albumin/creatinine ratio (ACR) >30 mg/g (43) were considered positives for kidney dysfunction. The glycosylated hemoglobin A1c (HbA1c) (CDA-VANTAGE) was measured at the enrolling time. Besides, the median of three or four HbA1c levels measurement one year before the enrolling time was used to evaluate the metabolic control status.

### Urine Sample Preparation

A morning urine sample (10–20 ml) was obtained from each patient. A protease inhibitors cocktail (Mini complex, Roche) was incorporated into each urine sample and then stored at −80°C. The urine was centrifuged at 17,000*g*, 15 min, 4°C). The pellet (cell fraction) was discharged and the supernatant (S1) was maintained at 4°C. Then, the S1 was ultracentrifuged at 200,000*g* for 1 h at 4°C to produce a pellet enriched in extracellular vesicles (E), and a supernatant free of extracellular vesicles (S). Both fractions were treated with lysis buffer (100 mM Tris–HCl, pH 6.8, 500 mM NaCl, 10% Tween 20, and protease inhibitor). Protein concentration was determined in each sample using the BCA kit (Pierce). Then, a 100 ug of total S or E protein fraction were analyzed by Western blot to establish the expression of NGAL, NFAT5, and HIF-1α.

### Extracellular Vesicles Identification

The presence of extracellular vesicles like exosomes was studied in both urine fractions (E and S) from T1DM and control group by measuring the Flotillin-1 (extracellular vesicle component) levels by Western blot (44) and confirmed by the standard protocol of transmission electron microscopy (TEM).

### Electrophoreses and Western Blot Analysis

The expression of Flotillin-1, NGAL, NFAT5, and HIF-1alpha were studied by Western blot according to standard conditions (45). Briefly, the proteins were separated on 7.5–10% polyacrylamide gels and transferred to nitrocellulose membranes (Invitrogen Carlsbad). Then, the membranes were blocked using 5% milk for 45 min at room temperature. Then, the membranes were probed with anti-Flotillin-1 (Abcam, Ab133497), anti-Lipocalin-2/NGAL (R & D Systems, Inc, MAB-1757), anti-NFAT5 (Thermo Scientific, PAI-023), or anti-HIF-1α (Abcam, Ab113642) antibodies overnight at 4°C. Following three washes with 0.1% Tween-20 in phosphate-buffered saline, the membranes were incubated with secondary [anti-mouse IgG-Alexa Fluor 750 (Thermo, A21037) or anti-rabbit IgG (Alexa Fluor 750, Thermo, A21039)] antibody in a 1:15,000 dilution for 2 h at room temperature. The infra-red fluorescence (IR) imaging was quantified using the Odyssey-CLx (Li-Cor) equipment and the software Image Studio Lite version 5.25 (Li-Cor).

### Statistical Analysis

Continuous variables are presented as median ± interquartile range (IQR). To Z-height/Age and Z-BMI/Age the results are expressed as mean ± SD. The Mann–Whitney test was used to compare all the continuous variables. To Z-height/Age and Z-BMI/Age t-Test was used. The categorical variables are expressed as frequency and Fisher’s exact test was used in the analysis. The correlation between variables was studied using a Spearman test. All data were processed in STATA version 12.0 and p-value <0.05 was considered statistically significant.

## Results

### Clinical and Biochemical Characterization of T1DM and Control Groups

Clinical data from T1DM (n = 34) and control (n = 15) groups are shown in **Table 1**. We did not find significant differences in sex (52.9 vs 33.3% men; p = 0.233), age (14.55 ± 5.50 vs 11.20 ± 4.50 years old; p = 0.070), Tanner stage, height/age Z score (−0.38 ± 0.93 vs −0.34 ± 1.25; p = 0.580), BMI Z score (0.66 ± 1.02 vs 0.91 ± 0.78; p = 0.390), frequency of systolic arterial hypertension (6/34 vs 1/15; p = 0.410), frequency of diastolic arterial hypertension (0/34 vs 2/15; p = 0.080) or median blood pressure (80.82 ± 9.68 vs 82.07 ± 8.47 mmHg; p = 0.500) in both groups.

**Table 1:** Clinical and biochemical characterization of T1DM and Control participants.

	T1DM n = 34	Control n = 15	p-value
**Sex (male/female)**	18/16	5/10	0.233
**Age (years)**	14.55 ± 5.50	11.20 ± 4.50	0.070
**Diagnostic Age (years)**	7.05 ± 7.40	–	–
**T1DM evolution (years)**	5.30 ± 6.50	–	–
**Z Height for age and sex**	−0.38 ± 0.93	−0.34 + 1.25	0.580
**Z BMI for age and sex**	0.66 ± 1.02	0.91 ± 0.78	0.390
**Tanner stage 1**	6	3	1.000
**2**	4	3	0.660
**3**	1	2	0.220
**4**	7	3	1.000
**5**	16	4	0.220
**SBP >p 95**	6	1	0.410
**DBP >p 95**	0	2	0.080
**MBP (mmHg)**	80.82 ± 9.68	82.07 ± 8.47	0.500
**HbA1c at enrollment time (%)**	8.60 + 2.70	–	–
**Median of HbA1c one year before enrollment (%)**	8.00 ± 2.80	–	–
**Serum Creatinine (mg/dl)**	0.56 ± 0.20	–	–
**MAU (mg/L)**	1.80 ± 11.40	–	–
**ACR mg/g**	2.83 ± 7.55	–	–
**GFR (ml/min/1.72 m^2^)**	165 ± 25	–	–
**MAU >20 mg/L**	4/34	–	–
**ACR >30 mg/g**	2/34	–	–
**GFR >140 ml/min/m^2^**	30/34	–	–
**NGAL-S (UA)**	8/34	0/15	**0.040**
**NGAL-E (UA)**	16/34	0/15	**0.001**
**NGAL-S (UA)**	3,355 ± 5,835	0	**0.040**
**NGAL-E (UA)**	7,495 ± 10,415	0	**0.002**
**HIF1α-S (UA)**	0	0	1.000
**HIF1α-E (UA)**	0	0	1.000
**NFAT5-S (UA)**	0	0	1.000
**NFAT5-E (UA)**	0	0	1.000
*NGAL-E vs NGAL-S			**0.020**

SBP, systolic blood pressure; DBP, diastolic blood pressure; MBP, mean blood pressure; HbA1c, glycosylated hemoglobin A1c; MAU, microalbuminuria; ACR, Albumin/creatinine urine ratio; GFR, Glomerular filtration rate by Schwartz formula; NGAL, neutrophil gelatinase-associated lipocalin; HIF-1α, Hipoxia-inducible factor-1 alpha subunit; NAFT5, Nuclear factor of activated T-cell 5; AU, Arbitrary Unit. Continuous variables are presented as median ± interquartile range (IQR). In the case of The Z height and Z BMI, the data are presented as mean ± SD. The Mann–Whitney test was used for all continuous variables, except for Z height and Z BMI in which t-test was used. The categorical variables are expressed as frequency and their analysis was used Fisher’s exact test. *NGAL-E vs NGAL-S considered only the samples with detectable levels of NGAL. Bold values means differences are statistically significant (p < 0.05).

In the T1DM group, the age at diabetes diagnosis was 7.05 ± 7.40 years old (1.0 to 14.5 years). The time from diabetes evolution was 5.30 ± 6.50 years (1.1 to 17.1 years). The median ± interquartile range of serum creatinine was 0.56 ± 0.20 mg/dl, MAU was 1.80 ± 11.40 mg/L, and ACR was 2.83 ± 7.55 mg/g (**Table 1**). The 11.8% (4/34) had MAU >20 mg/L, 5.9% (2/34) had ACR >30 mg/g, and 88.2% (30/34) had eGFR higher than 140 ml/min/1.72m^2^. The HbA1c at enrollment was 8.60 ± 2.70% and the median of HbA1c one year before was 8.00 ± 2.80%.

### Extracellular Vesicles Characterization

**Figure 1:**
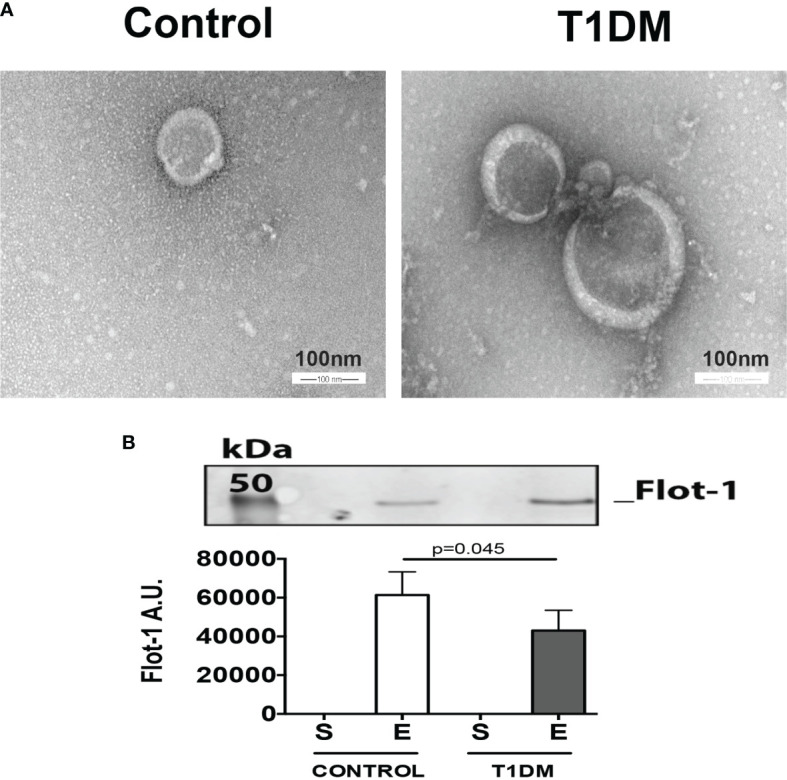
Extracellular vesicles are present in the urine samples of control and T1DM patients. **(A)** Representative figure of electron micrograph of isolated exosomes-like extracellular vesicles (scale bar, 100 nm). **(B)** Flot-1 levels in 100 mg of total protein of extracellular vesicle pellet. The upper panel is a representative picture of the Western blot of flotillin-1 (extracellular vesicle component). The graphic represents the median ± SEM of the levels of Flot-1 in the supernatant (S) and enriched extracellular fractions (E) generated by ultracentrifugation.

Extracellular vesicles were present only in the pellet after ultracentrifugation (E fraction) of urine in T1DM and control groups according to Western blot of flotillin (a well-characterized vesicle marker) and electronic microscopy (**Figure 1A**). Flotillin levels in T1DM were slight less in T1DM than control group (**Figure 1B**, p = 0.045), probably due to purification step by ultracentrifugation. Thus we normalized the expression of NGAL by total expression of proteins.

### NGAL, NFAT5, and HIF1α Expression

**Figure 2:**
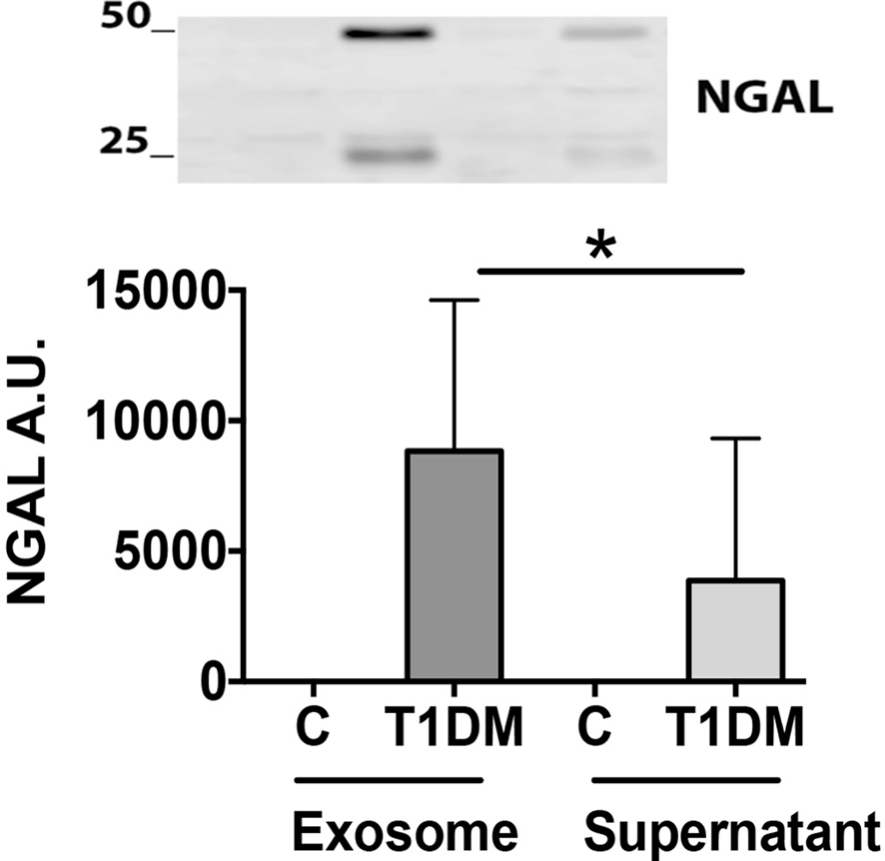
The NGAL level in urinary exosome enriched fraction is higher in T1DM than in control participants. Urine samples were processed to purify the extracellular vesicles by ultracentrifugation. The NGAL levels were studied by Western blot in the supernatant and the exosomes fraction (100 ug of total proteins). Upper panel representative picture of the two bands of NGAL (25 and 50 KDa). The lower panel is a graph with the NGAL signal. Arbitrary Unit (Median ± SEM; *p < 0.05).

The NGAL was detectable in 50% (17/34) of the T1DM group (positive NGAL-S or NGAL-E) but was undetectable in the urine (S or E fractions) from the control group (**Table 1**, **Figure 2**). The NGAL-E was present in 47% (16/34) and NGAL-S in 23.5% (8/34) of the T1DM group. On the other hand, 20.6% (7/34) had detectable NGAL expression in both fractions. Finally, 76.5% (13/17) of T1DM participants with detectable levels of NGAL (S or E fraction) had a normal MAU (lower than 20 mg/L).

**Figure 3:**
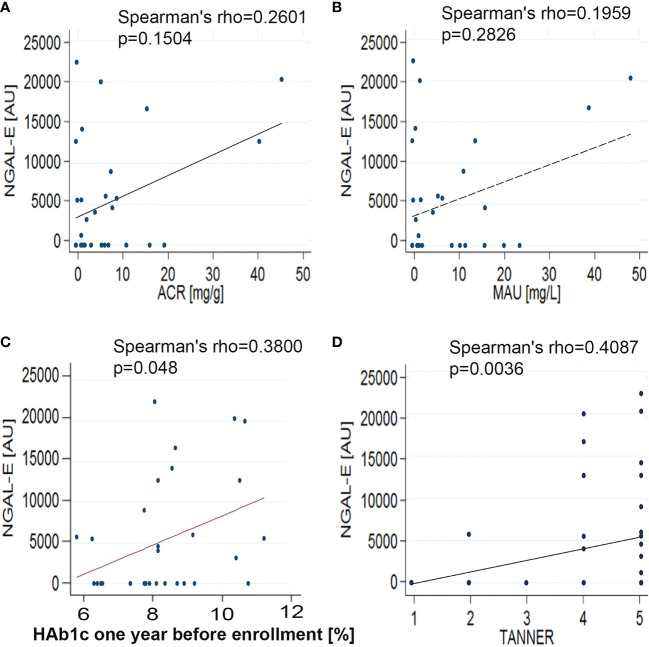
Correlation analysis between NGAL-E and kidney parameters. **(A)** NGAL vs ACR; **(B)** NGAL vs MAU; **(C)** NGAL vs HbA1c one year before enrollment, and **(D)** NGAL vs Tanner Stage. The correlation between variables was studied by Spearman test.

The NGAL-E was significantly higher than NGAL-S (NGAL-S: 3,355 ± 5,835 vs NGAL-E: 7,495 ± 10,415 AU/100 ug total protein; p = 0.02) (**Table 1** and **Figure 2**). NGAL-E correlated positively with Tanner stage (r = 0.41; p = 0.0036) and median of HbA1c one year before enrollment (r = 0.38; p = 0.048), but not with HbA1c at enrollment (r = 0.19; p = 0.25), ACR (r = 0.26; p = 0.15), or MAU (r = 0.19; p = 0.28) (**Figure 3**).

We did not find detectable levels of NFAT5 or HIF-1α in the E or S fraction in urine from both groups (T1DM and controls).

### Biochemical Characterization in Patients With Positive NGAL

Comparing positive (n = 17) and negative (n = 17) NGAL in T1MD group (E and/or S fractions) (**Table 2**) we found that positive NGAL subjects were older (15.60 ± 3.10 vs 12.00 ± 5.50 years, p = 0.03), had a longer diabetes evolution (6.60 ± 5.40 vs 4.34 ± 3.10 years, p = 0.03), higher serum creatinine levels (0.64 ± 0.10 vs 0.52 ± 0.12 mg/dl, p = 0.01), and higher median of HbA1c one year before enrollment (8.80 ± 3.00 vs 7.40 ± 1.30%, p = 0.045) than negative NGAL participants. NGAL was not observed with Tanner I pubertal stage (0/17 vs 6/17, p = 0.020) (**Table 2**). We observed a similar frequency of microalbuminuria (2/17 vs 2/17; p = 1.00), ACR (2/17 vs 0/17; p = 0.49), and hyperfiltration (GFR <140 ml/min/m^2^; 15/17 vs 15/17; p = 1.00) in positive and negative NGAL T1DM participants (**Table 2**). In addition, we did not find significant differences in the levels of microalbuminuria (1.90 ± 10.60 vs 1.50 ± 11.80; p = 0.39), ACR (4.17 ± 6.75 vs 1.90 ± 7.10; p = 0.230), or hyperfiltration (162 ± 27 vs 165 ± 21; p = 0.620) in both participants group (**Table 2**).

**Table 2:** Clinical and biochemical characterization of T1DM participant with positive NGAL and negative NGAL.

	NGAL (+) n = 17	NGAL (−) n = 17	p-value
**Sex (male/female)**	10/7	8/9	0.730
**Age (years)**	15.60 ± 3.10	12.00 ± 5.50	**0.030**
**Age at Diagnostic (years)**	8.00 + 8.00	7.00 + 5.00	0.640
**T1DM evolution (years)**	6.60 + 5.40	4.34 + 3.10	**0.030**
**Z Height for age and sex**	−0.34 ± 1.19	−0.42 ± 0.64	0.980
**Z IMC for age and sex**	0.76 ± 0.83	0.55 ± 1.20	0.570
**Tanner stage 1**	0	6	**0.020**
**2**	1	3	0.600
**3**	0	1	1.000
**4**	5	2	0.390
**5**	11	5	0.080
**SBP >p 95**	4/17	2/17	0.660
**DBP >p 95**	0	0	1.000
**MBP**	84.00 ± 9.81	78.00 ± 11.00	0.090
**HbA1c at enrollment (%)**	9.20 + 3.20	8.20 + 1.70	0.160
**Median of HbA1c one year before enrollment (%)**	8.80 ± 3.00	7.40 + 1.30	**0.045**
**Serum Creatinine (mg/dl)**	0.64 ± 0.10	0.52 ± 0.12	**0.010**
**MAU (mg/L)**	1.90 ± 10.60	1.50 ± 11.80	0.390
**ACR mg/g**	4.17 ± 6.75	1.90 ± 7.10	0.230
**GFR (ml/min/1.72 m^2^)**	162 ± 27	165 ± 21	0.620
**MAU >20**	2/17	2/17	1.000
**ACR >30**	2/17	0/17	0.490
**GFR >140 ml/min/m^2^**	15/17	15/17	1.000

SBP, systolic blood pressure; DBP, diastolic blood pressure; MBP, mean blood pressure; HbA1c, glicosylated hemoglobin A1c; MAU, microalbuminuria; ACR, Albumin/creatinine urine ratio; GFR, glomerular filtration rate by modified Schwartz formula for children. Continuous variables are presented as median ± interquartile range (IQR). In the case of The Z height and Z BMI, the data are presented as mean ± SD. The Mann–Whitney test was used for all continuous variables, except for Z height and Z BMI in which t-test was used. The categorical variables are expressed as frequency and their analysis was used Fisher’s exact test. Bold values means differences are statistically significant (p < 0.05).

## Discussion

Our results showed that NGAL, a biomarker of kidney tubular injury, is present in the urinary exosomal vesicles fraction in T1DM children and adolescents, suggesting its potential as an early biomarker of DKD. Interestingly, NGAL was detected only in T1DM children, with normal kidney parameters in some of them (MAU, ACR, and GFR), supporting the current idea that tubular damage is present in the early step of DKD (15, 16).

In the early stages of DKD, tubulointerstitial damage has been demonstrated by other methods. Histological changes at tubular and interstitial levels include hyperplasia and hypertrophy of tubular cells, followed by atrophy, tubular and peritubular dilatation, interstitial inflammation with mononuclear infiltration and glycogen accumulation. These findings appear even before glomerular changes and before microalbuminuria are detected (46–48). Multiple mechanisms have been involved as a cause of this tubular damage (49–51).

NGAL is considered a biomarker of tubular injury in the early stages of DKD and is likely good biomarker of this disease (52–54). Thus, current recommendations based on microalbuminuria for early detection of incipient DKD must be improved with the detection of tubulointerstitial injury biomarkers.

Here we show the first evidence that NGAL is present in enriched extracellular vesicles fractions obtained by ultracentrifugation of urine (47%; 16/34) from T1DM participants. We also found that the NGAL in the extracellular vesicles fraction was higher than the NGAL in urine without exosomes. In addition, NGAL in extracellular vesicles correlates with age, T1DM evolution, and serum creatinine levels, suggesting that extracellular vesicles could be used as a new source to detect early tubular damage during diabetic kidney disease.

NGAL is one of the most studied biomarkers of tubular damage, is secreted in high amounts into the urine and blood from tubular cells in acute kidney injury (AKI) even before creatinine rises (53, 54). In a NGAL mouse model locus, NGAL shows a sensitive, rapid, dose-dependent, reversible, organ, and cellular specific relationship with tubular stress (55).

In diabetic adolescents and adults, high levels of NGAL in blood and urine samples have been found before rising the microalbuminuria (42, 43). In addition, a positive correlation between NGAL, albumin excretion rate (AER), and glycated hemoglobin A1c (HbA1c) has been described in T2DM (22). Also, in T2DM, high levels of NGAL are predictive of impaired glomerular filtration rate (GFR) (50). Lacquaniti et al. (21), detected higher levels of urinary NGAL in T1DM than control (25.5 vs 6.5 ng/ml, p <0.0001; 50 T1DM and 35 controls). Similarly, Nielsen et al. (22) detected urinary NGAL levels significantly higher (146 vs 74 pg/mmol) in T1DM subjects (58 patients with normoalbuminuria and 55 controls). Additionally, a significant increase in urinary NGAL values was associated with increased albuminuria levels.

However, a few studies have investigated the expression of NGAL in children and adolescents. Papadopoulos et al. (56) studied serum NGAL in 57 T1DM children and 45 healthy participants, finding elevated levels of NGAL in the T1DM group (67.6 + 27.9 vs 24.6 + 15.8 ng/ml, p <0.001). Yuruk et al. (52) using centrifuged urinary samples (72 T1DM and 38 healthy children), found that urinary NGAL levels were significantly higher in T1DM compared to controls (100.16 ± 108.28 vs 21.46 ± 18.59 ng/ml; p = 0.0001). A positive correlation between urinary NGAL and MAU (r = 0.344, p = 0.002) was also described. Hafez et al. (57) found that urinary NGAL was present in 31.6% normoalbuminuric and 75% microalbuminuric patients, with a positive correlation between urinary NGAL and ACR, HbA1c, and time of T1DM evolution (50 children with T1DM for 5 or more years and 18 healthy controls).

Previous studies show that urinary extracellular vesicles better reflect the underlying protein, lipids, and mRNA changes in the kidney than whole urine samples (58). The exosomes-like extracellular vesicles allow a more certain knowledge of the internal metabolic cell status and have been used for diagnostic purposes in different diseases: pregnant cholestasis (59), risk of gestational diabetes mellitus (60), and kidney transplantation graft dysfunction (61, 62). The composition of molecules in the extracellular vesicles surface membrane and its content varies according to their cell of origin (63) allowing studying specific cell damage.

We previously, published that NGAL is present in the exosomal fraction of urine from kidney transplanted patients and it was associated with delayed graft function (62). Here we provided the first evidence to propose that exosomal-NGAL is a better early biomarker of DKD than free-NGAL in children and adolescents with T1DM. In our T1DM patients, 50% of children and adolescents had detectable NGAL (E and/or S fraction), 11% had MAU >20 mg/L, and 5.9% elevated ACR, suggesting that this tubular injury biomarker appears when the classic glomerular biomarkers of DKD are not present, this finding is agreeing to previous publication (22, 50). In the last decade, MAU has been questioned about its predictive value for DKD, because almost 50% of microalbuminuric patients regress to normoalbuminuria at 7 years follow-up (64, 65), and not all microalbuminuric patients progress gradually to ESRD (66). In addition, the non-albuminuric DKD phenotype has been described recently (67).

We found that NGAL in the enriched extracellular vesicles fraction was significantly higher than the supernatant fraction, similar to our previous results in delayed graft dysfunction in kidney transplantation (62). The latter supports the idea that extracellular vesicles are a new and important source to explore early tubular injury biomarker.

The NGAL in E and/or S fraction was higher in older participants with higher years of T1DM evolution and showed a positive correlation with the median of the HbA1c one year before enrollment, serum creatinine, and Tanner stage, but not with ACR, MAU, GFR, and HbA1 at enrollment, supporting previous findings that NGAL could be an early biomarker of DKD (46, 52).

Although most of the 17 with positive NGAL participants had a long evolution of T1DM, seven participants had less than 5 years of a T1DM diagnosis, suggesting that tubular compromise could appear in early states of T1DM. Interestingly, the MAU and ACR were normal in almost all the T1DM group, pointing out the relevance of having earlier biomarkers of kidney damage. Creatinine was normal in all T1DM participants, but hyperfiltration was present in 88% of T1DM participants. The high levels of HbA1c, with an estimated glucose level over 200 mg/dl, could explain the high incidence of hyperfiltration and as it has been described before, we did not find a correlation between NGAL and hyperfiltration (57).

In our study, we did not find a detectable signal of NGAL in the control group by Western blot in the S or E fractions. However, the literature has described detectable levels of NGAL in healthy controls by ELISA (52, 57). The potential explanation for these differences is the technique used (Western blot vs ELISA) and the antibody sensitivity to detect NGAL. Because we were interested in studying the extracellular vesicles fraction, we included two centrifugation steps: a regular centrifugation to eliminate cell fraction and ultracentrifugation to produce an S or E fraction. We used Western blot because this technique has been used to measure extracellular vesicles cargo for early detection of tubular injury in other kidney diseases and kidney transplantation grafts (44, 61, 62) and because there is not an ELISA kit for NGAL in the extracellular vesicle. We did not find other studies in diabetic participants using extracellular vesicle protein expression analysis.

Finally, we studied NFAT5 as a biomarker of hypertonicity and HIF-1α as a biomarker of hypoxia conditions because both molecules are closely related to diabetes (24, 30). Previously, we described that NFAT5 increase in kidneys subjected to renal ischemia and reperfusion injury in rats and mice (27, 68). In addition, NFAT5 had a role during diabetic complications such as atherosclerosis, diabetic nephropathy, and retinopathy (69). We have published the upregulation of Hif-1α in the kidney during renal ischemia and reperfusion injury in rats (68). Recently, the downregulation of glomerular filtration rate (GFR) observed during diabetic nephropathy was associated with Hif-1α. The Hif-1α upregulation may be involved in renal interstitial fibrosis in Diabetic Nephropathy (70). Here we described the first evidence intended to measure the levels of NFAT5 and Hif-1α in the soluble or extracellular vesicle fraction of urine. However, in our model, NFAT5 and HIF-1α expression was not detectable in the S or E fractions of urine (T1DM or control group). This suggests that NFAT5 and HIF-1α are not detectable by western blot in extracellular vesicles fraction of urine.

The limitations of our study are associated with a small sample size (34 T1DM and 15 controls), cross-sectional design, and only one-time determination. However, the strength of this study is to provide the first evidence that NGAL is present in the extracellular vesicles (exosomes) in the urine of DM individuals. In addition, NGAL-E is detectable when the free NGAL (not associated with extracellular vesicles) was undetected in urine. On the other hand, our findings allow us to propose the extracellular vesicles urine fraction as a new tool to identify early biomarkers of DKD in T1DM.

In conclusion, in our cross-sectional case-control study, NGAL was detectable in the urine fractions in 50% of children and adolescents with Type 1 diabetes mellitus, strongly suggesting tubular injury. In the control group, undetectable levels of NGAL were observed. We provide the first evidence that NGAL is present in the urinary enriched extracellular vesicles fraction (NGAL-E) in children and adolescent with T1DM and the NGAL levels were significantly higher than NGAL in urine without extracellular vesicles fractions (NGAL-S). Interestingly, NGAL-E did not correlated with albumin/creatinine ratio and HbA1c at time of enrollment. NGAL-E was present in patients without microalbuminuria or with normal albumin creatinine ratio, suggesting that NGAL extracellular vesicles precede classical parameters of early DKD. Therefore, we provided the first evidence to propose that exosomal-NGAL is a promising early biomarker of DKD and is better than free-NGAL in children and adolescents with T1DM. More studies are necessary to determine if NGAL-E has a predictive value for DKD progression and ESRD evolution.

## Data Availability Statement

The raw data supporting the conclusions of this article will be made available by the authors, without undue reservation.

## Ethics Statement

The studies involving human participants were reviewed and approved by the Scientific Ethics Committee from the South Metropolitan Public Health Service (Santiago-Chile). Written informed consent to participate in this study was provided by the participants’ legal guardian/next of kin.

## Author Contributions

FU and CEI conceived the experiments. VG, CG, AY, SV, and CS Collected sample. DS and JR Performed the experiments. FU, CEI, GC, PSM, and CU analyzed the data. FU, CEI, and FC helped perform the analysis with constructive discussions. FU and CEI contributed reagents, material and analysis tools. FU, CEI, and CP wrote the manuscript. All authors contributed to the article and approved the submitted version.

## Funding

FAI 292012 (Research Support Funds), Universidad de los Andes, Santiago, Chile). FONDECYT-1151157

## Conflict of Interest

The authors declare that the research was conducted in the absence of any commercial or financial relationships that could be construed as a potential conflict of interest.

## Publisher’s Note

All claims expressed in this article are solely those of the authors and do not necessarily represent those of their affiliated organizations, or those of the publisher, the editors and the reviewers. Any product that may be evaluated in this article, or claim that may be made by its manufacturer, is not guaranteed or endorsed by the publisher.
